# PReS-FINAL-2359: Renal involvement in hypocomplementemic urticarial vasculitis syndrome (huvs): report of 3 paediatric cases

**DOI:** 10.1186/1546-0096-11-S2-P349

**Published:** 2013-12-05

**Authors:** C Bracaglia, A Aceti, M Vivarelli, A Insalaco, M Pardeo, R Nicolai, F De Benedetti, A Pasini

**Affiliations:** 1 Division of Rheumatology, Department of Pediatric Medicine, IRCCS Ospedale Pediatrico Bambino Gesù, Rome, Italy; 2 Division of Nephrology and Dialysis, Department of Pediatric, Azienda Ospedaliera di Bologna, Bologna, Italy; 3 Division of Nephrology and Dialysis, IRCCS Ospedale Pediatrico Bambino Gesù, Rome, Italy; 4 Division of Nephrology and Dialysis, Azienda Ospedaliera di Bologna, Bologna, Italy

## Introduction

HUVS is a rare disease characterized by persistent urticarial lesions with histological evidence of leucocytoclastic vasculitis and complement activation with a marked decrease in circulating C1q levels. HUVS can present systemic features involving the musculoskeletal, pulmonary, renal and gastrointestinal systems; its peak incidence is in the fifth decade of life. The exact prevalence of HUVS in children is unknown.

## Objectives

We describe three children with HUVS with renal involvement.

## Methods

Three Caucasian children, two females and one male, presented, respectively at age of 9, 8 and 4 years, diffuse urticarial lesions, edema of lips, eyelids and feet, arthritis and arthralgia, abdominal pain and conjunctivitis. Two patients presented also a chronic restrictive respiratory insufficiency with numerous haemosiderin-laden macrophages at pulmonary biopsy.

## Results

At onset, in all three patients, autoimmunity (ANA, ANCA and anti-dsDNA) were negative, serum C3, C4 and C1q were low and anti-C1q antibodies were found to be markedly positive. Skin biopsy revealed leucocytoclastic vasculitis in all of them. Symptomatic therapy with antihistamines and non-steroidal anti-inflammatory drugs (NSAIDs) was ineffective. When dapsone was started skin involvement resolved completely. After approximately 4 years from onset the three children presented a renal involvement: one with persistent microhaematuria, one with persistent microhematuria and proteinuria and one with significant proteinuria. A renal biopsy showed three different histological findings: mesangial glomerulonephritis with membranous features, mesangial glomerulonephritis associated with focal necrotizing small-vessel vasculitis and glomerulonephritis with intense mesangial, endo- and extra-capillary proliferation with initial tubular atrophy. The first child was treated with oral glucorticoid and mycophenolate mofetil, the other two with pulses of intravenous metilprednisolone and cyclophosphamide. All three patients presented a good clinical response.

## Conclusion

HUVS affects primarily female adults and only 8 paediatric cases have been described. In our patients renal involvement was not present at onset and urinary abnormalities appeared later during disease course. HUVS is rare in children, renal involvement is probably more frequent and more severe than in adults and may appear later. Isolated microhematuria can be the only sign of a subclinical renal involvement: its role should not be underestimated and renal biopsy should be considered. Reports of rapid progression to end-stage renal disease suggest that prompt treatment is necessary. Dapsone is the recommended therapy however its efficacy on renal involvement is not known.

## Disclosure of interest

None declared.

